# Isochromosome Mosaic Turner Syndrome With Concomitant Hypopituitarism and Multiple Meningiomas

**DOI:** 10.7759/cureus.66548

**Published:** 2024-08-09

**Authors:** T. Saideekshit, Meenakshi Sundari S. N., Siva Govindan, Shiva Prakash, M. Radhika

**Affiliations:** 1 Department of Internal Medicine, Sri Ramaswamy Memorial (SRM) Medical College Hospital and Research Centre, SRM Institute of Science and Technology, Kattankulathur, IND

**Keywords:** hypogonadotropic hypogonadism, karyotyping, meningioma, isochromosome mosaic turner syndrome, turner syndrome

## Abstract

Isochromosome mosaic Turner syndrome (IMTS) is a rare genetic variant of Turner syndrome (TS). The diagnosis of TS can be missed until adolescence or early adulthood in females with minimal symptoms. The clinical features of mosaic TS can be atypical and should be evaluated thoroughly to detect potential complications. Here, we describe a unique report of a 47-year-old woman diagnosed with IMTS, hypogonadotropic hypogonadism, and multiple meningiomas. She presented with decreased responsiveness and decreased appetite. She had primary amenorrhea, hearing loss, and visual impairment for which focused medical care was not sought. Physical examination revealed short stature, short neck, Tanner stage 3 breast, Tanner stage 1 vaginal development, and absent axillary and pubic hair, which led us to a clinical diagnosis of TS. A transabdominal ultrasound revealed a hypoplastic uterus with no visualized ovaries. A slit lamp examination revealed bilateral immature cataracts and optic atrophy. An audiogram confirmed sensorineural hearing loss. The intelligence quotient was below average. Hormonal assays showed hypogonadotropic hypogonadism and secondary adrenal insufficiency, which is not a feature of TS. This abnormal hormonal assay prompted us to do magnetic resonance imaging of the brain, which showed meningiomas in the suprasellar region and left cerebellopontine angle. Karyotyping revealed 46,X,i(X)(q10)(37)/45,X(3), which was suggestive of IMTS. The patient required a multidisciplinary approach in the evaluation, diagnosis, and management, which included hormone replacement therapy and supportive and psychological care.

## Introduction

Isochromosome mosaic Turner syndrome (IMTS) is characterized by the presence of 45,X, with other cells showing 46,XX, and also the coexistence of isochromosome, i.e., the presence of a structurally faulty X chromosome with two short arms (Xp) or two long arms (Xq) in some cell lines [1]. This even rarer entity, represented as 45,X/46,X,i(X)(q10), is only 8%-9% prevalent in women with Turner syndrome (TS) [2]. In contrast to the most prevalent variety of TS (45,X or monosomy X), the symptoms of IMTS are less severe and can vary [3].

Clinical features can be atypical and should be investigated thoroughly. In the 46,X,i(Xq) karyotype, which has an isochromosome on the Xq, short stature is the common feature. On the other hand, 46,X,i(Xp), which has an isochromosome on the Xp of the X chromosome and is a very rare entity, gonadal dysfunction is more likely to occur [4]. The diagnosis of TS may not be made until later in life, such as in childhood when a short stature evaluation suggests the diagnosis, in adolescence when growth failure and delayed puberty raise the possibility of TS, or even in adulthood when the diagnosis is discovered during an infertility or amenorrhea workup [5]. Patients with IMTS can develop complications such as autoimmune thyroid disease, sensorineural hearing loss, malformed kidneys, bicuspid aortic valve, coarctation of the aorta, and mental retardation [2]. The management of IMTS includes early clinical suspicion, especially given the atypical presentations, diagnosis of the condition, hormone replacement therapy, and screening and treatment of complications to enhance the quality of life.

Classically, the affected females have hypergonadotropic hypogonadism. The case described here is unique, as karyotyping and hormonal assay suggested IMTS with hypogonadotropic hypogonadism, along with associated suprasellar and left cerebellopontine (CP) angle meningiomas on magnetic resonance imaging (MRI).

This case study describes a 47-year-old female with primary amenorrhea, short stature, visual impairment, and hard of hearing, diagnosed as IMTS with hypogonadotropic hypogonadism and multiple meningiomas. This report emphasizes the significance of karyotyping in the early diagnosis of TS presenting with atypical features, as the phenotypic expression is variable with regard to different karyotypes. This also signifies the importance of a holistic approach and appropriate referral in the further management of patients with TS for prompt detection and prevention of complications.

## Case presentation

A 47-year-old woman presented to our outpatient department clinic with her father, complaining of decreased appetite and responsiveness. She was short, measuring 144 cm and weighing 42 kg. Her body mass index was 20.3 kg/m^2^, and her vital signs were unremarkable. Capillary blood glucose was 66 mg/dL. Physical examination revealed the patient had a short neck with a low posterior hairline, a broad chest with bilateral Tanner stage 3 breast development, and no axillary or pubic hair.

On further questioning, the patient’s father revealed that she had delayed developmental milestones, particularly gross motor milestones like standing (achieved at three years old). She also suffered from hard of hearing for six years and visual impairment for five years but never sought medical attention for these conditions. She had not attained menarche. She was a single child who was born out of a nonconsanguineous marriage via an uneventful normal vaginal home delivery. She lost her mother during childhood due to a medical condition that the attendees could not recollect. They were from a remote village where the father was practicing herbal medicine. According to him, no antenatal screening was performed nor did they seek modern medicine at any point in her life for any of her symptoms until this presentation. The patient took herbal medicines for delayed puberty, which was ineffective. There were no known familial medical conditions or a history of short stature or delayed puberty among family members or relatives. She was unmarried with no history of sexual contact. She denied deleterious habits or substance abuse. She finished high school in their village, where her performance was average.

Visual impairment mandated an ophthalmological evaluation, which revealed bilateral immature cataracts and optic atrophy. Otoscopic examination showed bilateral grade 3 tympanic membrane retraction. Hearing tests and a pure tone audiogram revealed sensorineural hearing loss. Genital examination revealed a blind vagina (Tanner stage 1) and absent pubic hair. Cardiopulmonary and abdominal examinations were unremarkable. There were no palpable inguinal masses. The Glasgow Coma Scale was E4V5M6 and had no focal neurological deficits. The cerebellar examination was normal. The history and physical examination led to the provisional diagnosis of TS, which prompted the planning of an evaluation to confirm the diagnosis and search for associated features.

The patient's laboratory values included hemoglobin 11 g/dL, total leucocyte count 6,400/mm^3^, platelet count 225,000/mm3, random blood sugar 70 mg/dL, sodium 124 mEq/L, potassium 2.8 mEq/L, bicarbonate 22 mEq/L, calcium 9 mg/dL, creatinine 0.6 mg/dL, and urea 12 mg/dL. Liver function and fasting lipid profile were within normal limits.

The levels of follicle-stimulating hormone, luteinizing hormone, growth hormone (GH), and cortisol were low, whereas the prolactin level was normal. Adrenocorticotropic hormone levels were also found to be low, which indicated secondary adrenal insufficiency. Thyroid-stimulating hormone and free T4 were within normal limits (Table 1).

**Table 1:** Hormonal assay FSH: follicle stimulating hormone; LH: luteinizing hormone; HRT: hormone replacement therapy; GH: growth hormone; ACTH: adrenocorticotropic hormone; TSH: thyroid stimulating hormone; ng: nanogram; mL: milliliter; mIU: milli-international units; mcg: microgram; dL: deciliter; pg: picogram

Hormone	Patient’s lab values	Standard reference values
Prolactin	5.8 ng/mL	5-23 ng/mL
FSH	0.4 mIU/mL	Normal menstruating women: 1.38-16.69 mIU/mL; postmenopausal women: 26.7-136.4 mIU/mL
LH	0.02 mIU/mL	Normal menstruating women: 0.56-89.08 mIU/mL; postmenopausal women without HRT: 5-62 mIU/mL
Cortisol	2.1 mcg/dL	Morning: 3.7-19.4 mcg/dL
GH	0.12 ng/dL	Females: up to 8 ng/dL
ACTH	<5 pg/mL	7.2-63.3 pg/mL
TSH	2.30 mIU/mL	0.5–4 mIU/mL
Free T4	1.2 ng/dL	0.58–1.64 ng/dL

The hormonal assay in this patient was a captivating revelation because it indicated hypogonadotropic hypogonadism, which is not an expected finding in TS, which typically has hypergonadotropic hypogonadism.

**Figure 1:**
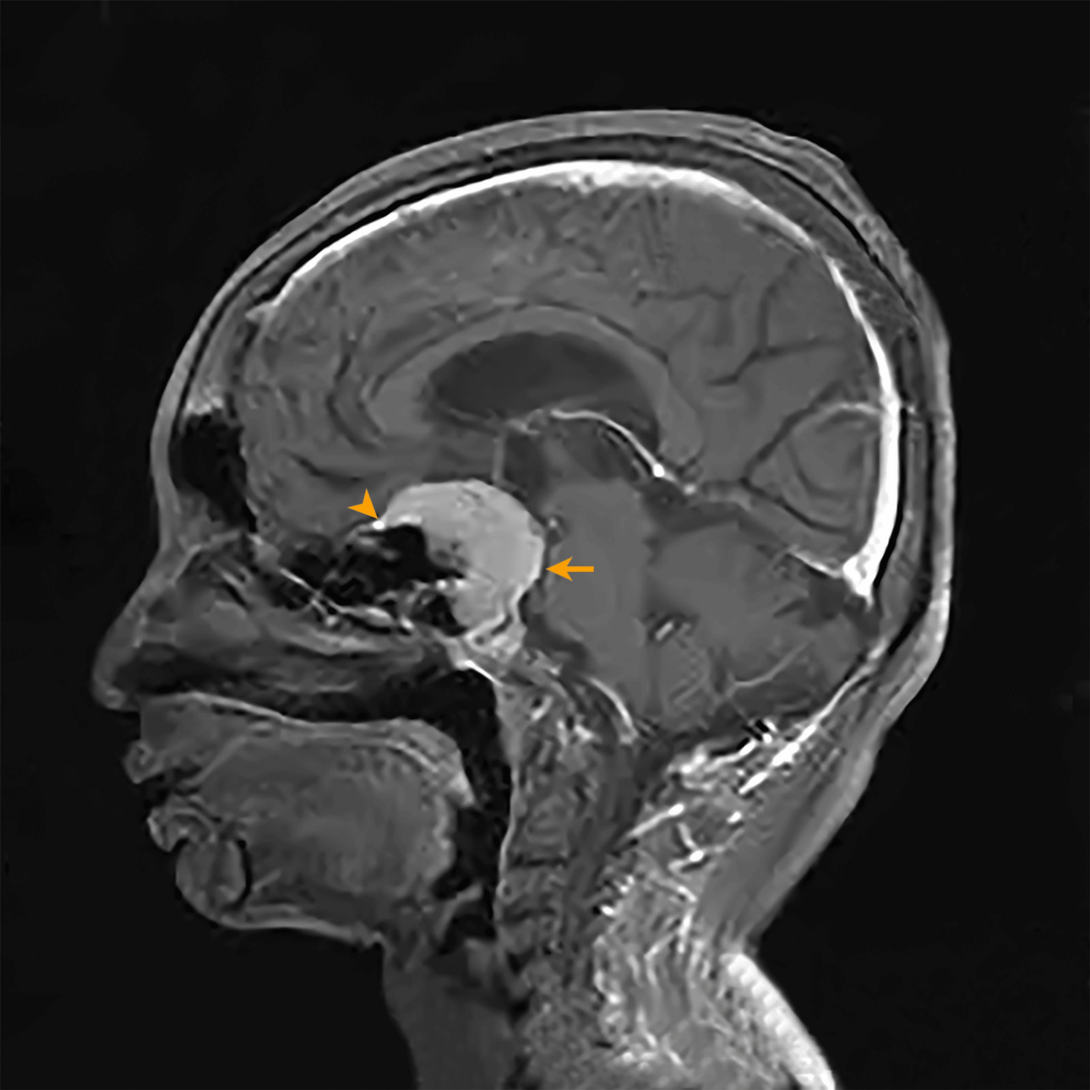
MRI gadolinium-enhanced T1-weighted sagittal image. A well-defined enhancing lesion of 4.3 x 3.9 x 3.6 cm in the suprasellar extending into the sella (orange arrow). The lesion shows a dural tail sign (orange arrowhead), suggesting a meningioma. There is no separate visualization of the pituitary gland MRI: magnetic resonance imaging

**Figure 2:**
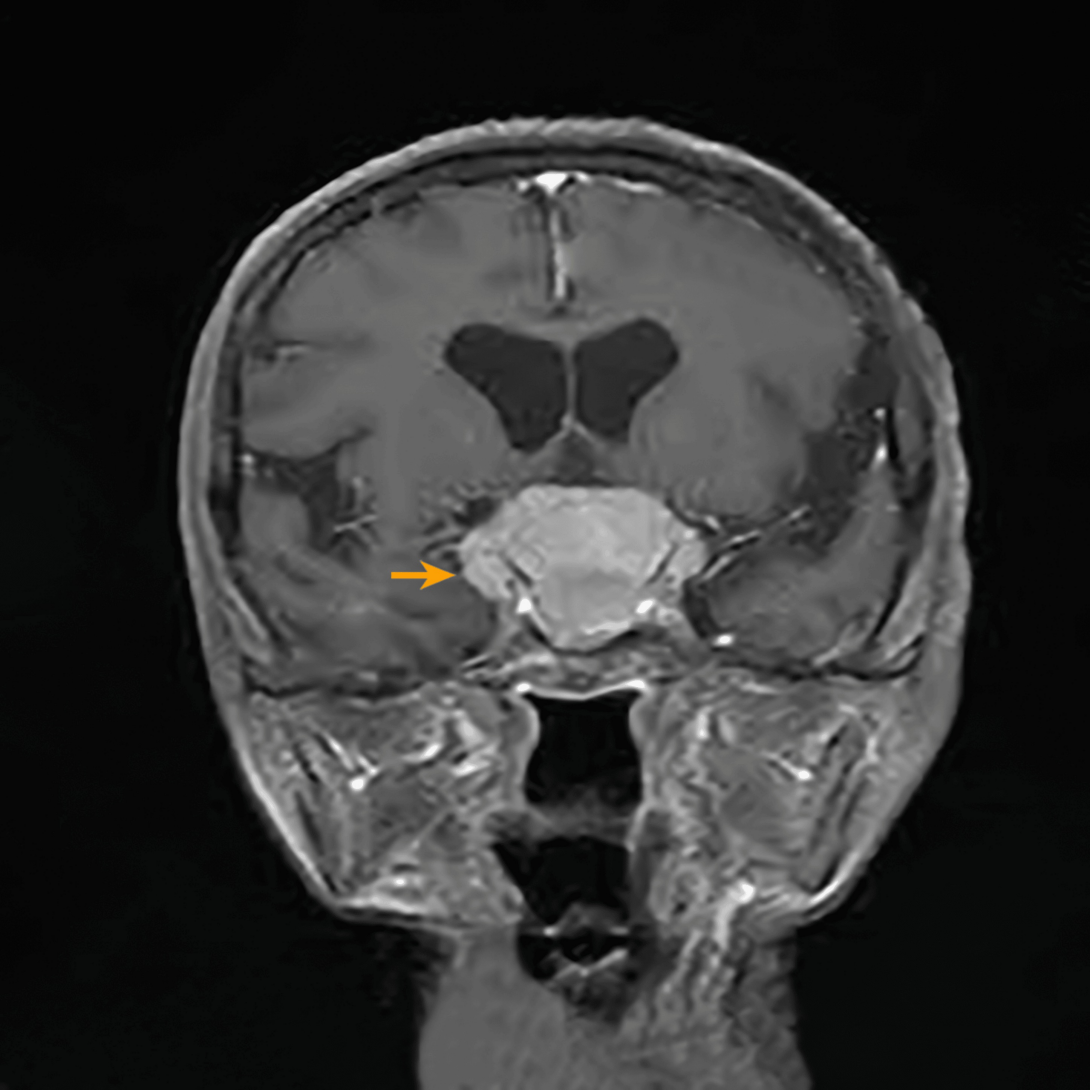
MRI gadolinium-enhanced T1-weighted coronal image, showing the tumor (orange arrow) extending anteriorly into the bilateral optic canal and planum sphenoidale region MRI: magnetic resonance imaging

This necessitated an evaluation for hypothalamo-pituitary lesions. MRI brain revealed a meningioma in the suprasellar region that extended into the sellar region, resulting in pituitary fossa enlargement and splaying of the anterior clinoid process (Figures 1, 2).

**Figure 3:**
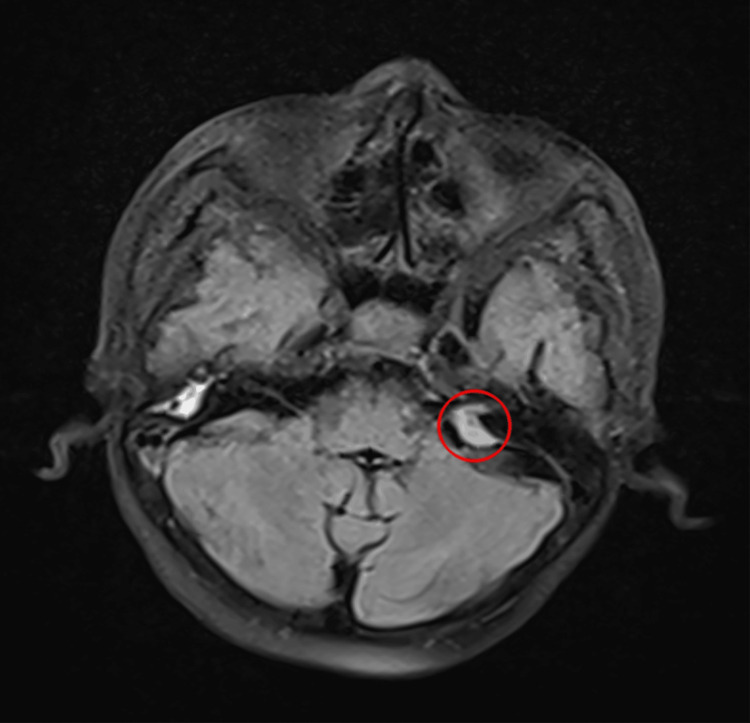
MRI gadolinium-enhanced T1-weighted axial image, showing a hypointense lesion (within the red circle) of 1.2 x 0.9 x 0.9 cm in the left CP angle extending into the left internal acoustic canal MRI: magnetic resonance imaging; CP: cerebellopontine

The lesion extended anteriorly into the bilateral optic canal and planum sphenoidal region. The left CP angle revealed a second meningioma (Figure 3).

**Figure 4:**
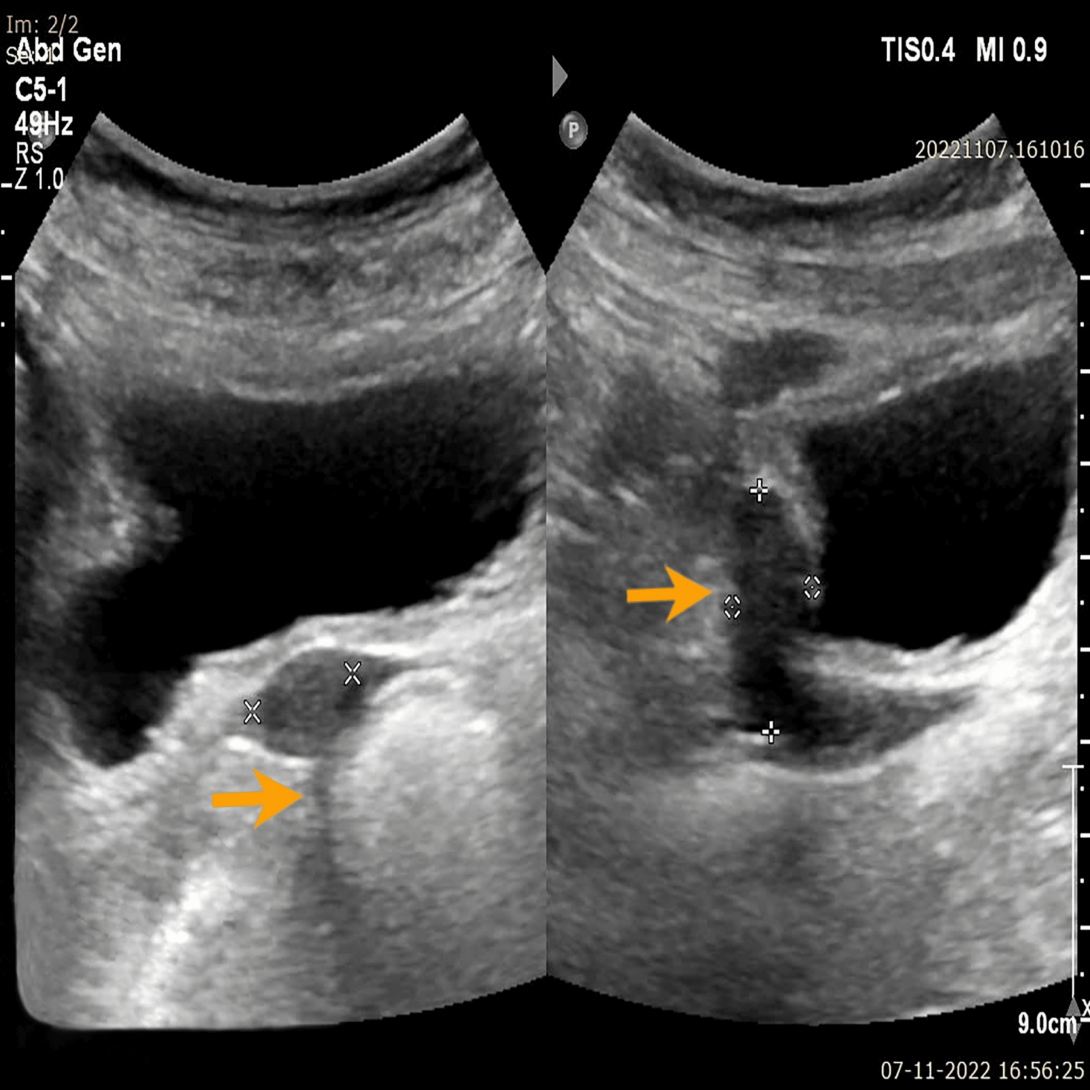
Ultrasound pelvis, showing hypoplastic uterus (orange arrows) with no visualized ovaries

Ultrasonography of the pelvis showed a hypoplastic uterus, whereas the ovaries could not be visualized (Figure 4).

**Figure 5:**
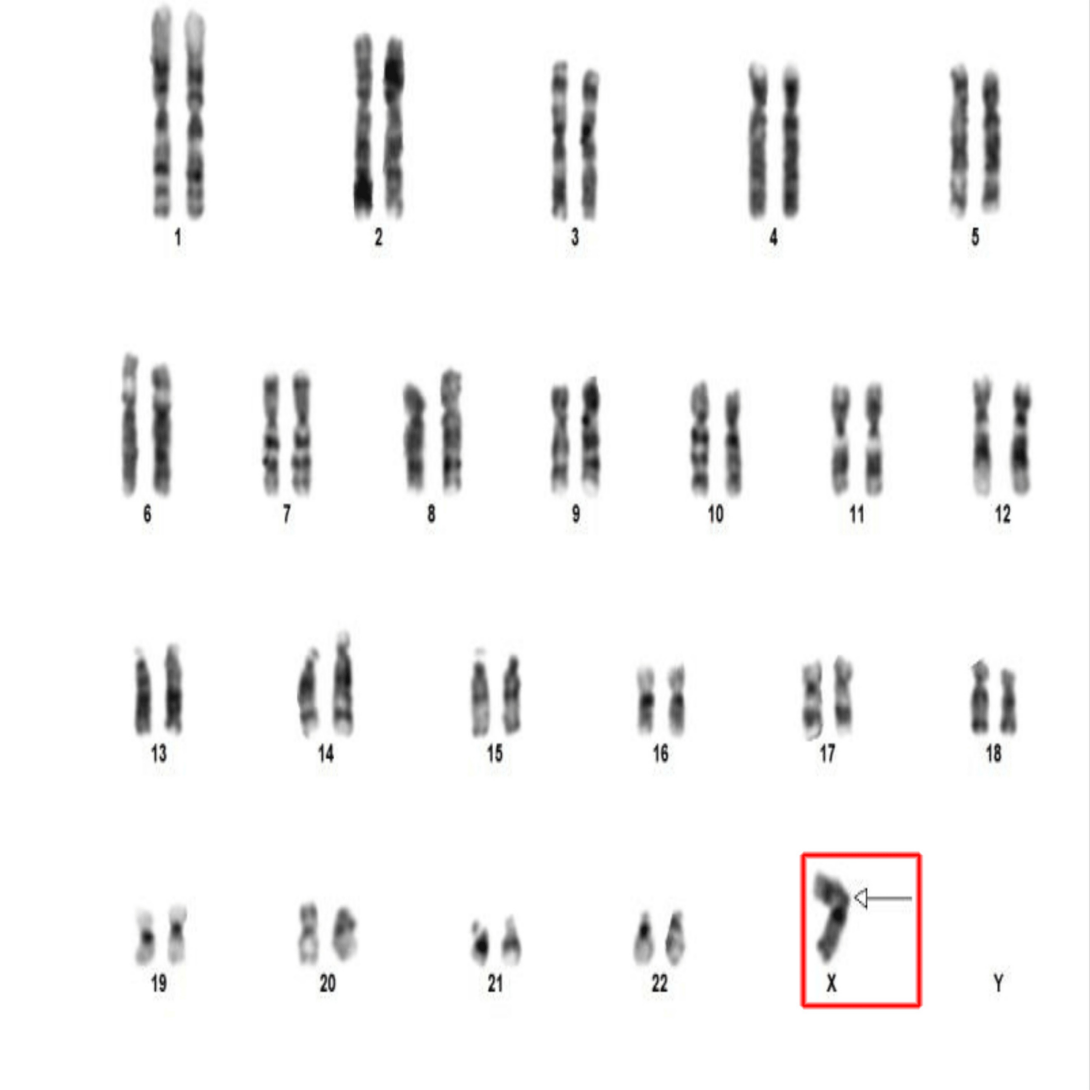
Karyotyping image at 450-550 bphs resolution showing monosomy X (45,X) (red box) bphs: bands per haplotype set

After radiological investigations, we attributed the hormonal abnormalities to suprasellar meningioma, which extends into the sella and causes hypopituitarism. 2D echocardiography showed normal findings. An ultrasound of the kidneys was unremarkable. Karyotyping of peripheral blood via the G-banding technique was performed, and 40 cells were examined at a band resolution of 450-550 bands per haplotype set. Three cells revealed the presence of only one X chromosome, 45,X (monosomy X) (Figure 5).

**Figure 6:**
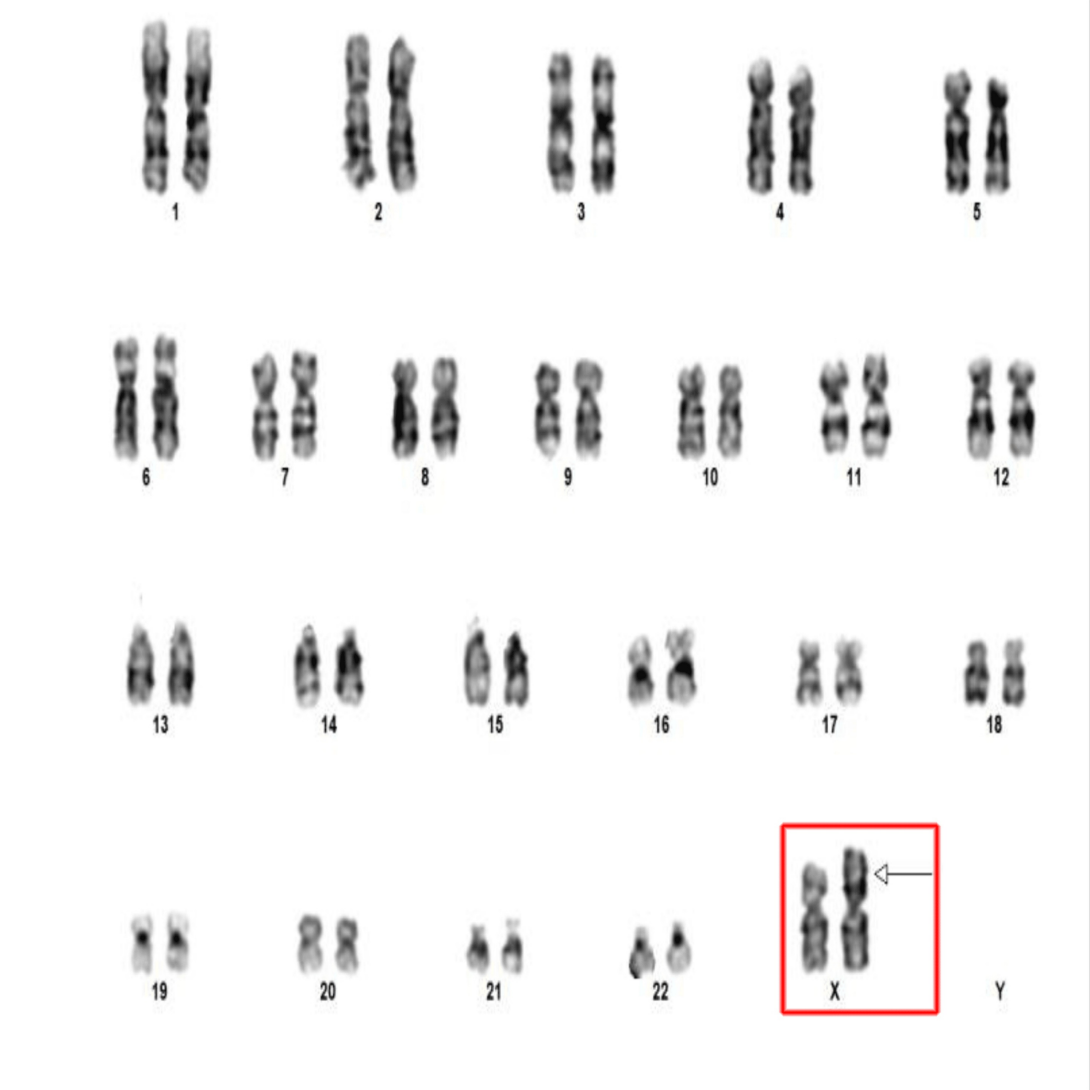
Karyotyping image at 450-550 bphs resolution showing isochromosome of the X chromosome (red box) with an arrow pointing toward the long arm bphs: bands per haplotype set

The other 37 cells showed an isochromosome of the X chromosome in the Xq at band 10 (Xi)(q10) (Figure 6).

Her karyotype, 46,X,i(X)(q10)(37)/45,X(3) was thus diagnosed as an IMTS variant.

## Discussion

A female with TS, also known as the 45,X, has a partial or whole loss of an X chromosome. One in 2,500 to one in 3,000 female newborns [1] are affected by this disorder of sexual development [6]. There are various TS variations according to the karyotype, with monosomy X being the most prevalent. Others include deletions of Xq or Xp, ring chromosome formations, isochromosome formation (Xi), and mosaicism, in which a monosomy X (45,X) variant is associated with one or more cell lines with a defective X chromosome.

This case underscores the complexity and the diverse clinical presentation of TS, particularly when diagnosis is delayed. TS is typically identified early in life due to its characteristic features and developmental concerns. However, this patient's presentation at 47 years of age, without prior medical intervention and associated suprasellar and CP angle meningiomas, is exceptionally rare and can pose diagnostic and management challenges. Karyotype in this instance showed 46,X,i(X)(q10)/45,X. It is a mix of mosaic and isochromosome patterns. The occurrence of such a Turner karyotype is only 8%-9% [1].

Stunted growth/short height, gonadal dysgenesis, distinctive facial features, webbing of the neck, shield-like chest with widely spaced nipples, low posterior hairline, nevi throughout the body, short metacarpals and fingernails, improper development of the breast, and cardiovascular and renal anomalies are among the frequently observed features of TS. Nonetheless, most of these symptoms differ significantly based on the karyotypic variant. It has been found that individuals with mosaicism for the 46,XX or X isochromosome have milder symptoms [7]. Likewise, some of the common signs were absent in our patient.

Among women with TS, short stature is the most easily identifiable feature. Short stature homeobox-containing gene (SHOX), which is essential for the normal development of the skeleton, is haplo-insufficient (present only as one copy, whereas two copies are needed normally), which results in the height defect. Because SHOX gene expression is primarily located around the elbow, knee, and wrist joints, structural bone abnormalities account for a large portion of the physical features associated with TS, in addition to short stature [8]. The hallmark of TS, hypergonadotropic hypogonadism, is due to ovarian failure and the absence of pituitary feedback inhibition. Partial or total loss of the X chromosome in the germ cells causes ovarian stromal fibrosis and rapid oocyte degeneration in TS patients. Our patient's lack of secondary sexual features can be attributed to ovarian failure. However, our patient had hypogonadotropic hypogonadism, which is not a characteristic finding in TS and is attributed to suprasellar meningioma extending into the sella, thereby suppressing the pituitary function.

The association of TS with hypopituitarism is unusual and noteworthy. Hypopituitarism in TS can result from various etiologies, with pituitary adenoma, empty sella, and cranial irradiation among the reported causes. To date, only a few reports are available in the literature regarding TS and hypogonadotropic hypogonadism. Those patients had thalassemia major, hemochromatosis [9], autoimmune disorder, combined immunodeficiency [10], or empty sella as their cause. Typically, meningiomas constitute 1%-3% of the sellar tumors removed via transsphenoidal surgery [11]. The presence of a suprasellar meningioma complicating TS has rarely been reported. Currently, 13 cases of meningiomas coexisting in TS patients have been published in the literature [12], of which only five were multiple meningiomas.

To our knowledge, this is the first case report with IMTS associated with hypogonadotropic hypogonadism and multiple meningiomas. Women with TS seem to be at higher risk for meningioma and other brain tumors, among other malignancies [13,14].

Cardiovascular problems, which affect 29% of TS patients with isochromosome mosaic patterns, are particularly associated with increased mortality in TS patients. The most prevalent congenital cardiac defect is the bicuspid aortic valve, which can coexist with other abnormalities like coarctation of the aorta [15]. Our patient's two-dimensional echocardiogram did not show any of these abnormalities. The incidence of ischemic heart disease, when compared to the general population, is twice as high for people who have TS [7] due to dyslipidemia [16]. Among TS patients, 25%-30% have hypothyroidism, which is caused by an autoimmune thyroid condition [3]. The thyroid profile for our patient was within normal limits and can be attributed to the varied presentation of IMTS.

Patients with TS also frequently have structural abnormalities of the kidneys and ears. Our patient had no renal abnormalities on ultrasound, whereas otoscopy and audiogram revealed bilateral grade 3 retracted tympanic membrane and bilateral sensory neural hearing loss, respectively. However, the size of the left CP angle meningioma in our case is too small to cause neurological symptoms. The absence of tinnitus and vertigo probably explains the benign nature of this CP-angle tumor.

Sensorineural hearing loss, characterized by a sensorineural drop in the 1.5-2 kHz range, sensorineural high-frequency hearing loss, or all of these, affects most women with TS (50%-90%) [17]. TS is often associated with ocular abnormalities like ptosis, hypertelorism, and epicanthal folds. In addition, red-green color impairment is prevalent in TS. Among TS children, 25%-35% had hyperopia and nonfamilial strabismus. Children with TS should be examined by a pediatric ophthalmologist between the ages of 12 and 18 months to prevent visual loss [17]. Case reports also mention keratoconus, glaucoma, cataracts, anterior lenticonus, and retinal neovascularization as additional characteristics [18,19]. Our patient had bilateral optic atrophy secondary to suprasellar meningioma extending anteriorly into the optic canal. Due to bilateral optic atrophy and immature cataracts in both eyes, our patient had severe visual impairment. It is unclear if many of the ocular diseases addressed have a true link with TS or represent associations by chance because of the small number of cases in the literature [20].

Most female TS patients have a normal intelligence quotient (IQ), except in cases of mosaic variant, especially IMTS, where 9% of patients exhibit mental retardation. The IQ of our patient, measured with a standardized tool, ranged between 70 and 79, which is equivalent to a person whose mental capacity is below average.

Following diagnosis and treatment of the medical issues (hyponatremia, hypokalemia, and hypoglycemia), hormone replacement therapy was the goal of management, along with health education and counseling. For secondary adrenal insufficiency, a three-day intravenous injection of hydrocortisone 100 mg was administered, followed by an oral course of hydrocortisone 30 mg/day in divided doses, which was gradually tapered. A neurosurgery opinion was sought regarding meningiomas and advised transsphenoidal surgical removal of the tumor. However, we deferred the surgery because the patient and her caregivers failed to provide informed consent.

Considering the financial restraints, the biological age of our patient, and the hypoplastic uterus, GH and sex hormone replacements were deferred. She was supplemented with vitamin D3, calcium, and magnesium, along with dietary advice. Following treatment, the patient's overall health progressively improved, and there were no further episodes of hyponatremia, hypokalemia, or hypoglycemia, and the patient's decreased responsiveness was attributed to hyponatremia or hypoglycemia. Long-term plans included monitoring and appropriate titration of hormone replacement therapy.

## Conclusions

This case report shows that multiple meningiomas and TS do not usually occur together. In addition, the coexistence of hypogonadotropic hypogonadism and the IMTS subtype in the same patient makes this case report unique, thus validating its rationale for being reported here. It has been shown by this report that there are multiple variations of TS based on their karyotype, and the symptoms of isochromosome mosaicism are milder than those of the more prevalent pure 45,X variant. We recommend karyotyping studies and MRI/CT brain imaging for all individuals suspected of having TS, particularly if their presentation is atypical. This case report has also demonstrated how crucial a multidisciplinary approach is, with extensive evaluation and screening, right from the point of diagnosis to promptly detect and prevent possible complications.
